# Practical diagnosis and treatment of premenstrual syndrome and premenstrual dysphoric disorder by psychiatrists and obstetricians/gynecologists in Japan

**DOI:** 10.1002/pcn5.234

**Published:** 2024-08-15

**Authors:** Kana Yoshimi, Fumi Inoue, Tamami Odai, Nahoko Shirato, Zen Watanabe, Tempei Otsubo, Masakazu Terauchi, Takashi Takeda

**Affiliations:** ^1^ Division of Women's Health, Research, Institute of Traditional Asian Medicine Kindai University Osaka Japan; ^2^ Department of Psychiatry Kyowakai Hannnan, Hospital Osaka Japan; ^3^ Department of Women's Health Tokyo Medical and Dental University Tokyo Japan; ^4^ Department of Obstetrics and Gynecology Showa University School of Medicine Tokyo Japan; ^5^ Department of Obstetrics and Gynecology Tohoku University Graduate School of Medicine Miyagi Japan; ^6^ Department of Psychosomatic and Psychiatric Medicine Tokyo Women's Medical University Adachi Medical Centerr Tokyo Japan

**Keywords:** diagnostic techniques and procedures, Kampo, oral contraceptive pills, prospective daily charting, selective serotonin reuptake inhibitors

## Abstract

**Aim:**

To investigate and compare the diagnoses and treatment of premenstrual syndrome (PMS) and premenstrual dysphoric disorder (PMDD) from the perspectives of psychiatrists and obstetricians/gynecologists (OB/GYNs) in Japan.

**Methods:**

Between December 2021 and February 2022, a web‐based survey was conducted among the members of the Japanese Association of Neuro‐Psychiatric Clinics. Data from 262 psychiatrists who responded to the aforementioned survey were compared with data from 409 OB/GYNs from a survey conducted in 2021 among members of the Japanese Society of Obstetrics and Gynecology.

**Results:**

Overall, 79.8% of psychiatrists and 97.3% of OB/GYNs were involved in practicing PMS/PMDD diagnosis and treatment. Most psychiatrists believed that PMS should be treated by OB/GYNs (74.4%) and PMDD by psychiatrists (75.6%). Only vague medical interviews were conducted by 86.6% of psychiatrists, and only 9.7% maintained a two‐cycle symptom diary. Psychiatrists mostly prescribed selective serotonin/serotonin and noradrenaline reuptake inhibitor (SSRI/SNRI) continuous dosing (91.1%), followed by Kampo medicines, especially *Kamishoyosan* (73.3%); only 2.8% chose oral contraceptive pills, unlike OB/GYNs, while SSRI continuous (32.8%) and luteal phase dosing (20.6%) and Kampo medicine (42.1%) were the most common first‐line treatments. Lifestyle guidance was prescribed by 63.6% of psychiatrists, followed by cognitive behavioral therapy (13.8%) and the symptom diary observation method (11.1%), which were similar to OB/GYNs' choices.

**Conclusions:**

Many Japanese psychiatrists and OB/GYNs do not base PMS/PMDD diagnoses on prospective monitoring methods using specific diagnostic criteria and therefore do not provide evidence‐based treatment. Moreover, a tendency of being biased toward treatments in which the department specialized was observed.

## Graphical abstract

This study aimed to clarify the current status and problems associated with premenstrual syndrome/premenstrual dysphoric disorder (PMS/PMDD) diagnosis and treatment by psychiatrists and compare them with those by obstetricians/gynecologists. The results indicate that most psychiatrists in Japan based their diagnosis of PMS/PMDD on vague medical interviews rather than on a prospective monitoring method according to the relevant diagnostic criteria. A tendency for bias toward treatments in which their department specializes was observed, which differs from treatments in accordance with global standard guidelines, and many treatments are unique to Japan, such as Kampo medicine.

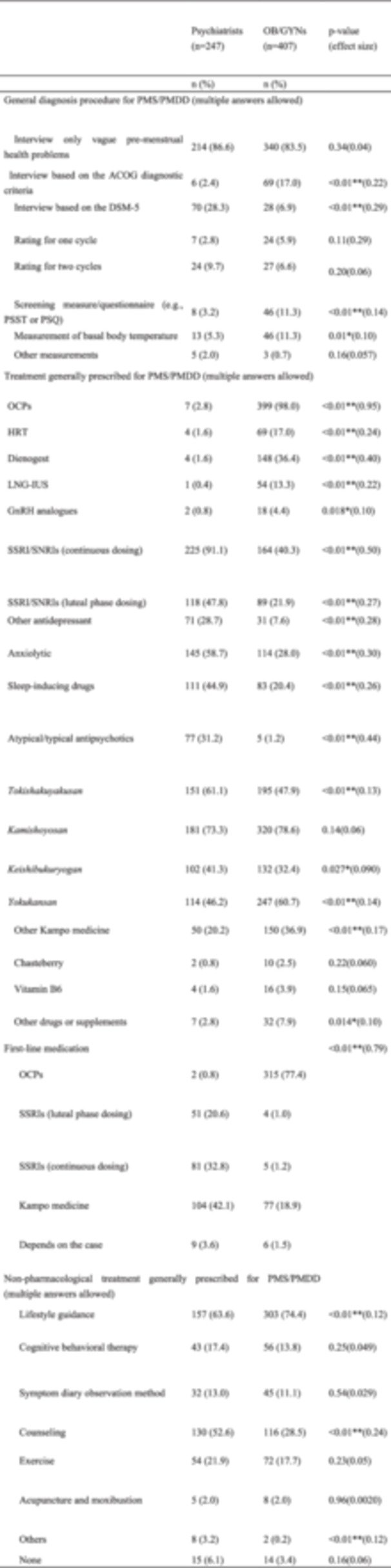

## INTRODUCTION

Premenstrual syndrome (PMS) and premenstrual dysphoric disorder (PMDD) are characterized by psychiatric or physical/behavioral symptoms that manifest during the luteal phase of the menstrual cycle, affect patients' activities of daily living, and resolve shortly after the onset of menstruation. Affective symptoms include affective lability, irritability, depressed mood, and anxiety. Physical/behavioral symptoms include decreased interest, difficulty in concentration, anergia, cravings or changes in appetite, insomnia or hypersomnia, a sense of being overwhelmed or out of control, and physical symptoms such as breast tenderness or swelling, joint or muscle pain, bloating or weight gain.^1^, ^2^, ^3^

Epidemiological surveys have estimated that the frequency of premenstrual symptoms is relatively high (80%–90%) and that the prevalence of PMS in menstruating women is approximately 20%–30%. Furthermore, 1.2%–6.4% of women of reproductive age exhibit severe psychotic premenstrual symptoms associated with PMDD that interfere with daily life.^1^, ^4^, ^5^, ^6^

PMS and PMDD are classified as premenstrual conditions from the perspective of gynecology and psychiatry, respectively, and although the established diagnostic methods are different, there is some overlap between them. The American Psychiatric Association has defined and published specific criteria for a severe clinical syndrome of PMDD in the Diagnostic and Statistical Manual of Mental Disorders, Fifth Edition, Text Revision (DSM‐5‐TR).^3^ Similarly, the American Congress of Obstetricians and Gynecologists (ACOG) has defined guidelines for PMS.^7^ Nevertheless, there has been considerable controversy and unevenness in guidelines across disciplines about the appropriate diagnostic criteria for syndromes with clinically significant premenstrual symptoms. Notably, PMS and PMDD have recently been viewed as a part of the continuous disease concept. The International Society for the Study of Premenstrual Disorders (ISPMD) describes a spectrum of premenstrual disorders (PMDs), including core and variant PMDs, and both PMS and PMDD are considered core PMDs.^8^ The DSM‐5‐TR,^3^ ISPMD, and ACOG guidelines^7^, ^8^ state that symptoms must occur reproducibly during two cycles of prospective recording for a diagnosis of PMS and PMDD. As a prospective symptom diary, the Daily Record of Problem Severity (DRSP) is considered a valid and reliable tool to diagnose PMS or PMDD and is frequently used for research purposes, including in clinical trials worldwide.^9^, ^10^ However, while screening tools and structured clinical interviews are available and are clinically used, such retrospective assessments are often considered to be limited because of their subjectivity and concerns regarding recall bias. This is because patients can overestimate the cyclical nature of their symptoms, which are irregular and often worse during the luteal phase.^1^, ^8^ Moreover, 2 months of prospective daily symptom monitoring requires a considerable amount of time and effort from patients, therefore its adoption in routine clinical practice remains questionable. In 2021, we conducted a survey to investigate the current status and problems associated with diagnosing and treating PMS/PMDD among Japanese obstetricians and gynecologists (OB/GYNs). The results showed that only 8.4% of the 1267 OB/GYN respondents engaged in routine PMS/PMDD treatment used a two‐cycle symptom diary.^11^ This was similar to the percentage (11.5%) reported for a 2012 study that included gynecologists and family physicians in the United States,^12^ indicating a dissociation between research and actual clinical practice.

Standard pharmacological treatments for PMS/PMDD include the use of selective serotonin reuptake inhibitors (SSRIs), which affect the levels of serotonin and other neurotransmitters in the brain, and oral contraceptive pills (OCPs), which influence hormonal activity by suppressing ovulation. SSRI therapy, dosed continuously or only in the luteal phase of the menstrual cycle, is the gold standard treatment for PMDD as per expert guidelines.^13^, ^14^ Serotonin and norepinephrine reuptake inhibitors (SNRIs) such as venlafaxine have shown to be therapeutically effective for PMDD.^15^ For treating women with PMS, drospirenone‐containing OCPs may represent an effective treatment and should be considered as the first‐line pharmaceutical intervention OCPs.^10^, ^16^ The guidelines of the Japanese Society of Obstetrics and Gynecology (JSOG) state that treatment can also include counseling, lifestyle guidance, exercise therapy, and administration of Kampo medicines (traditional Japanese herbal medicines) and diuretics.^17^

Notwithstanding these recommendations, insurance does not cover SSRIs and OCPs for patients with PMS/PMDD in Japan. Moreover, in addition to the medical complications, both these drug types are associated with several other complications. OCPs have side effects such as the increased risk of venous thromboembolism, stroke, and breast cancer, and SSRIs can increase suicide‐related behavior in patients younger than 24 years, activation, and postdiscontinuation symptoms.^18^, ^19^, ^20^ In this context, according to the results of the survey of OB/GYNs in 2021, OCPs were the most common first‐line drugs for the treatment of PMS/PMDD (76.8%), following Kampo medicine (19.5%) and SSRIs, which were significantly less frequently used (2.6%).^11^

PMS/PMDD is often treated by OB/GYNs and psychiatrists in Japan, and the definition of the disease concept has not been fully established. Due to the variety of disease concepts and diagnosis methods for PMS/PMDD, deciding which department a patient should be referred to remains a significant problem. It is assumed that psychiatrists and OB/GYNs also face various challenges in diagnosing and treating PMS/PMDD; however, the actual situation is unclear. The present study therefore aimed to clarify the current status and problems associated with the diagnosis and treatment of PMS/PMDD in Japan by psychiatrists and to compare them with those by OB/GYNs.

## METHODS

### Ethical considerations

This study was performed as a Women's Health Care Academic Committee survey of the JSOG and targeted physicians who belong to the JSOG and the Japanese Association of Neuro‐Psychiatric Clinics (JAPC). This study was conducted in accordance with the principles of the Declaration of Helsinki. The survey was conducted anonymously and contained no personal information. A description of the study's objectives was provided to all participants and they agreed to participate by submitting online consent.

### Participants

**Figure 1:**
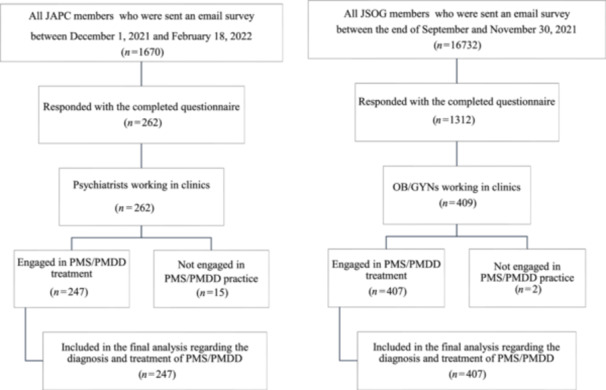
Participant selection flowchart. JAPC, Japanese Association of Neuro‐Psychiatric Clinics; JSOG, Japanese Society of Obstetrics and Gynecology; OB/GYNs, obstetricians/gynecologists; PMS, premenstrual syndrome; PMDD, premenstrual dysphoric disorder.

A survey questionnaire was mailed to all JSOG members (*n* = 16,732) between the end of September and the end of November 2021. The details of the survey have been reported previously.^11^ A survey questionnaire was also emailed to all JAPC members (*n* = 1670) between December 1, 2021 and February 18, 2022, and a web‐based survey was administered using Google Forms. Of these, 262 JAPC members responded (15.7%) with the completed questionnaires. Among them, 247 psychiatrists who routinely engaged in PMS/PMDD treatment and provided answers to questions about their PMS/PMDD diagnosis and treatment were selected (Figure 1). The results of this survey have already been outlined in JSOG's annual report.^21^ Of the 1312 members of the JSOG who responded to the survey, 409 OB/GYNs (2.4% of all JSOG members [16,732]) whose place of work was a clinic were selected because all JAPC members work in clinics. Among them, 407 who engaged in routine PMS/PMDD treatment and answered the questions regarding routine PMS/PMDD diagnosis and treatment were included in this study.

### Questionnaire

With regard to general characteristics, participants were questioned about the number of years since licensure as doctors and their gender. We also surveyed their knowledge about disease names, diagnosis, and treatment, which department (OB/GYN, psychiatry, internal medicine, or others) should diagnose and treat PMS and PMDD according to them, and the frequency of involvement. Only those engaged in PMS/PMDD practice were questioned about their routine diagnostic procedure for PMS/PMDD, which first‐line drugs and pharmacotherapies they used most commonly in the treatment of PMS/PMDD, and which non‐pharmacotherapy treatments they used most frequently. Multiple answers were allowed for the question regarding which department should diagnose and treat PMS and PMDD, and regarding treatment and diagnosis in accordance with the previous survey for OB/GYNs in 2021.^11^ “Interview based on the DSM‐5” was included in the answers to the question on “General diagnostic procedure for PMS/PMDD.”

The DSM‐5‐TR published in 2022 maintained the same diagnostic criteria for PMDD without specific changes, compared to the DSM‐5.^3^, ^22^

### Statistical analysis

Cross‐tabulations were performed, and Pearson's *χ* ^2^ tests were conducted between psychiatrists and OB/GYNs. Statistical significance was set at *P* < 0.05. The effect size was measured using Cramer's *V* calculated with BellCurve for Excel (Social Survey Research Information Co., Ltd). The effect sizes of 0.10, 0.30, and 0.50 were judged as small, medium, and large, respectively.^23^

## RESULTS

The background characteristics of participants are listed in Table 1. The gender ratio (female vs. male) was 19.5 versus 80.5 in psychiatrists, and 41.8 versus 57.5 in OB/GYNs. The number of male psychiatrists was significantly higher than that of female psychiatrists. The postlicensure period was significantly longer for the psychiatrists than for the OB/GYNs.

**Table 1:** Characteristics of study participants.

	Psychiatrists (*n* = 262)	OB/GYNs (*n* = 409)	*P* value (effect size)
	*n* (%)	*n* (%)	
*Postlicensure period (years) for medical practitioners*			0.014* (0.15)
<10	2 (0.8)	4 (1.0)	
≥10 and <20	19 (7.3)	58 (14.2)	
≥20 and <30	70 (26.7)	128 (31.3)	
≥30 and <40	95 (36.3)	139 (34.0)	
≥40 and <50	70 (26.7)	73 (17.8)	
≥50	6 (2.3)	7 (1.7)	
*Gender*			<0.01** (0.24)
Female	51 (19.5)	171 (41.8)	
Male	211 (80.5)	235 (57.5)	
No response	0 (0)	3 (0.7)	

Abbreviation: OB/GYNs, obstetricians/gynecologists.

* *P* < 0.05

** *P* < 0.01.

Table 2 presents data regarding the involved degree of engagement in and awareness of PMS/PMDD care. Notably, 79.8% of psychiatrists (*n* = 262) answered “practice on diagnosis and treatment of PMS/PMDD.” Significantly more psychiatrists (60.3%) chose psychiatry as the preferred treatment approach for PMS, whereas only 32.0% of OB/GYNs shared this perspective. Conversely, for PMDD, 77.8% of OB/GYNs indicated a preference for treatment within the OB/GYN specialty, a significantly higher proportion compared to the 63.4% of psychiatrists who expressed the same opinion. In total, 247 (94.3%) psychiatrists and 407 (99.5%) OB/GYNs answered that they were engaged in routine PMS/PMDD treatment; OB/GYNs treated PMS/PMDD more frequently than psychiatrists.

**Table 2:** Knowledge about and involvement in PMS/PMDD diagnosis and treatment.

	Psychiatrists (*n* = 262)	OB/GYNs (*n* = 409)	*P* value (effect size)
	*n* (%)	*n* (%)	
*Knowledge about PMS/PMDD diagnosis and treatment*			<0.01* (0.32)
No knowledge	2 (0.8)	5 (1.2)	
Knowledge of name only	36 (13.7)	3 (0.7)	
Knowledge of diagnosis and treatment	15 (5.7)	3 (0.7)	
Practicing diagnosis and treatment	209 (79.8)	398 (97.3)	
*Departments that should diagnose and treat PMS (multiple answers allowed)*			
Psychiatry	158 (60.3)	131 (32.0)	<0.01* (0.28)
Gynecology	195 (74.4)	382 (93.3)	<0.01* (0.26)
Internal medicine	17 (6.5)	20 (4.9)	0.48 (0.034)
Either	67 (25.6)	34 (8.3)	<0.01* (0.24)
*Departments that should diagnose and treat PMDD (multiple answers allowed)*			
Psychiatry	198 (75.6)	295 (72.1)	0.37 (0.038)
Gynecology	166 (63.4)	318 (77.8)	<0.01* (0.16)
Internal medicine	8 (3.1)	12 (2.9)	0.92 (0.0034)
Either	48 (18.3)	40 (9.8)	<0.01* (0.12)
*Engaged in PMS/PMDD treatment*			<0.01* (0.47)
No	15 (5.7)	2 (0.5)	
Rarely (a few patients per year)	66 (25.2)	18 (4.4)	
Occasionally (a few patients per month)	113 (43.1)	99 (24.2)	
Daily (several patients per week)	68 (26.0)	290 (70.9)	

Abbreviations: OB/GYNs, obstetricians/gynecologists; PMS/PMDD, premenstrual syndrome and premenstrual dysphoric disorder.

* *P* < 0.01.

The results of the questions concerning the diagnosis and treatment of PMS/PMDD are presented in Table 3. Regarding the generic diagnostic procedures, 86.6% of psychiatrists answered “Interview only vague premenstrual health problems,” similar to the result for OB/GYNs (83.5%). Only 9.7% of psychiatry respondents maintained a symptom diary rating for two cycles, as described by the ACOG and DSM‐5‐TR diagnostic criteria.^3^, ^7^ These findings did not differ significantly from those of the OB/GYNs. Some OB/GYNs choose an ACOG‐based interview (17.0%), while psychiatrists chose a DSM‐5‐based interview (28.3%). Additionally, psychiatrists (3.2%) used a screening questionnaire like the Premenstrual Symptoms Screening Tool or Premenstrual Symptoms Questionnaire^24^, ^25^, ^26^, ^27^ significantly less often than OB/GYNs (11.3%).

**Table 3:** Responses regarding the diagnosis and treatment of PMS/PMDD.

	Psychiatrists (*n* = 247)	OB/GYNs (*n* = 407)	*P* value (effect size)
	*n* (%)	*n* (%)	
*General diagnosis procedure for PMS/PMDD (multiple answers allowed)*			
Interview only vague premenstrual health problems	214 (86.6)	340 (83.5)	0.34 (0.04)
Interview based on the ACOG diagnostic criteria	6 (2.4)	69 (17.0)	<0.01** (0.22)
Interview based on the DSM‐5	70 (28.3)	28 (6.9)	<0.01** (0.29)
Rating for one cycle	7 (2.8)	24 (5.9)	0.11 (0.29)
Rating for two cycles	24 (9.7)	27 (6.6)	0.20 (0.06)
Screening measure/questionnaire (e.g., PSST or PSQ)	8 (3.2)	46 (11.3)	<0.01** (0.14)
Measurement of basal body temperature	13 (5.3)	46 (11.3)	0.01* (0.10)
Other measurements	5 (2.0)	3 (0.7)	0.16 (0.057)
*Treatment generally prescribed for PMS/PMDD (multiple answers allowed)*			
OCPs	7 (2.8)	399 (98.0)	<0.01** (0.95)
HRT	4 (1.6)	69 (17.0)	<0.01** (0.24)
Dienogest	4 (1.6)	148 (36.4)	<0.01** (0.40)
LNG‐IUS	1 (0.4)	54 (13.3)	<0.01** (0.22)
GnRH analogues	2 (0.8)	18 (4.4)	0.018* (0.10)
SSRI/SNRIs (continuous dosing)	225 (91.1)	164 (40.3)	<0.01** (0.50)
SSRI/SNRIs (luteal phase dosing)	118 (47.8)	89 (21.9)	<0.01** (0.27)
Other antidepressant	71 (28.7)	31 (7.6)	<0.01** (0.28)
Anxiolytic	145 (58.7)	114 (28.0)	<0.01** (0.30)
Sleep‐inducing drugs	111 (44.9)	83 (20.4)	<0.01** (0.26)
Atypical/typical antipsychotics	77 (31.2)	5 (1.2)	<0.01** (0.44)
Tokishakuyakusan	151 (61.1)	195 (47.9)	<0.01** (0.13)
Kamishoyosan	181 (73.3)	320 (78.6)	0.14 (0.06)
Keishibukuryogan	102 (41.3)	132 (32.4)	0.027* (0.090)
Yokukansan	114 (46.2)	247 (60.7)	<0.01** (0.14)
Other Kampo medicine	50 (20.2)	150 (36.9)	<0.01** (0.17)
Chasteberry	2 (0.8)	10 (2.5)	0.22 (0.060)
Vitamin B_6_	4 (1.6)	16 (3.9)	0.15 (0.065)
Other drugs or supplements	7 (2.8)	32 (7.9)	0.014* (0.10)
*First‐line medication*			<0.01** (0.79)
OCPs	2 (0.8)	315 (77.4)	
SSRIs (luteal phase dosing)	51 (20.6)	4 (1.0)	
SSRIs (continuous dosing)	81 (32.8)	5 (1.2)	
Kampo medicine	104 (42.1)	77 (18.9)	
Depends on the case	9 (3.6)	6 (1.5)	
*Nonpharmacological treatment generally prescribed for PMS/PMDD (multiple answers allowed)*			
Lifestyle guidance	157 (63.6)	303 (74.4)	<0.01** (0.12)
Cognitive behavioral therapy	43 (17.4)	56 (13.8)	0.25 (0.049)
Symptom diary observation method	32 (13.0)	45 (11.1)	0.54 (0.029)
Counseling	130 (52.6)	116 (28.5)	<0.01** (0.24)
Exercise	54 (21.9)	72 (17.7)	0.23 (0.05)
Acupuncture and moxibustion	5 (2.0)	8 (2.0)	0.96 (0.0020)
Others	8 (3.2)	2 (0.2)	<0.01** (0.12)
None	15 (6.1)	14 (3.4)	0.16 (0.06)

Abbreviations: ACOG, the American College of Obstetricians and Gynecologists; DSM‐5, the Diagnostic and Statistical Manual of Mental Disorders‐5; GnRH‐analogue, gonadotropin‐releasing hormone agonists and antagonists; HRT, hormone replacement therapy; LNG‐IUS, levonorgestrel intrauterine system; OB/GYNs, obstetricians/gynecologists; OCPs, oral contraceptives; PMS/PMDD, premenstrual syndrome and premenstrual dysphoric disorder; PSQ, the Premenstrual Symptoms Questionnaire; PSST, the Premenstrual Symptoms Screening Tool, SSRI/SNRI, selective serotonin reuptake inhibitors/serotonin noradrenaline reuptake inhibitors.

* *P* < 0.05

** *P* < 0.01.

SSRI/SNRI continuous dosing was the most commonly prescribed drug therapy by psychiatrists (91.1%). In contrast, the percentage for SSRI/SNRI–luteal phase dosing was 47.8%. Kampo medicine (especially *Kamishoyosan* [73.3%], *Tokishakuyakusan* [61.1%], and *Yokukansan* [46.2%]) was the next most common treatment. Psychiatrists more frequently prescribed psychotropic drugs, for example anti‐anxiety agents (58.7%), sleep‐inducing drugs (44.9%), and atypical/typical antipsychotics (31.2%). In contrast, hormone therapy was much less commonly chosen by psychiatrists compared to OB/GYNs.

Regarding the first‐line drugs for PMS/PMDD treatment, psychiatrists chose Kampo medicine (42.1%), followed by SSRI/SNRI continuous dosing (32.8%) and SSRI/SNRI luteal phase dosing (20.6%); only 0.8% chose OCPs. Significantly more psychiatrists chose Kampo medicine than OB/GYNs.

Lifestyle guidance was chosen by 63.6% of psychiatrists as the most common non‐pharmacological treatment, following counseling (52.6%), exercise guidance (21.9%), cognitive behavioral therapy (CBT) (17.4%), and the symptom diary observation method (13.0%).

## DISCUSSION

This study aimed to identify the current PMS/PMDD diagnosis and treatment practices among psychiatrists and compare them with previously published results for OB/GYNs in Japan. PMS/PMDD and other menstruation‐related disorders are often treated by psychiatrists and OB/GYNs in Japan, and according to this survey, many practicing psychiatrists and OB/GYNs were treating PMS/PMDD.

Both groups of practitioners had overwhelmingly vague opinions regarding diagnostic methods (86.6% of psychiatrists and 83.5% of OB/GYNs), with only 28% of psychiatrists using the DSM‐5 and only 17% of OB/GYNs using the ACOG diagnostic criteria. Very few (9.7% of psychiatrists and 6.6% of OB/GYNs) had an assessment of prospective two consecutive menstrual cycles based on the diagnostic criteria for PMS/PMDD. This indicates that there is a discrepancy between the various diagnostic criteria and actual clinical practice. However, the findings of our study closely align with those of a 2012 US study that reported a similar prevalence rate of 11.5%,^12^ although it is important to note that the previous study focused specifically on OB/GYN and family medicine physicians, while our research encompassed all OB/GYNs, including those working in hospital settings, in Japan.^28^ According to the DSM‐5‐TR, women with psychiatric disorders other than PMS/PMDD may experience chronic or intermittent symptoms that are not related to the menstrual cycle; however, the onset of menstruation is likely to be a memorable event, leading to complaints that symptoms worsen or only appear during the premenstrual period. The differential diagnosis is further complicated by the overlap of symptoms between PMDD and several other psychiatric diagnoses. This challenge is particularly pronounced when clinicians depend solely on recalled symptoms, therefore it is crucial to confirm PMDD through prospective rating scales.^3^

Although diagnoses based on prospective assessments such as the DRSP are necessary for research objectives, the diagnostic methods proposed by these guidelines are inconsistent with clinical practice.^9^ The proportion of OB/GYNs using the screening tool was low (11.3%) and for psychiatrists was even lower (3.2%). The reason for the significant difference between them is unclear, but it could be the fact that this tool is not well known in both departments. Therefore, it is questionable whether many psychiatrists are making accurate diagnoses, and further education of psychiatrists regarding PMS/PMDD diagnosis appears to be needed. Future studies are required to establish clinically appropriate and convenient diagnostic procedures and biomarkers that can be used to measure the severity of the disease quantitatively.

Regarding treatment drugs when multiple responses were allowed, SSRI/SNRI (continuous dosing) was 91.1%, SSRI/SNRI (luteal phase dosing) was 47.8%, followed by Kampo medicines (Kamishoyosan: 73.3%), whereas only 2.8% administered OCPs among psychiatrists. These results were in the reverse order for OB/GYNs. Furthermore, as for first‐line treatment, most chose SSRIs (53.2% [continuous dosing 32.8%, luteal phase dosing 20.6%]), followed by Kampo medicines (42.1%). Psychiatrists and OB/GYNs also had contrasting choices for this question. Regarding pharmacotherapy, there was a tendency for treatment to be biased toward the speciality of each department. Regarding the high proportion of psychiatrists administering SSRIs, it is assumed that a high proportion of them treat patients with severe psychiatric symptoms, which makes this the correct treatment method, as indicated in the treatment guidelines for PMDD.^10^, ^13^ As for SSRI administration methods, luteal phase administration alone is as effective as continuous administration.^29^ The present results suggest that psychiatrists may be unaware of this fact, as many of them use continuous dosing as their first treatment choice.

This study also showed that although the frequency of SSRI use was relatively high (53.2%) in psychiatry, Kampo medicine was also preferred (42.1%) as a first‐line drug. This may be due to concerns regarding the side effects of SSRIs.^20^ Kampo medicines are more common as first‐line treatment, which means that many practitioners are making treatment choices contrary to the guidelines for PMS/PMDD. Both departments chose a variety of Kampo medications that are universally used in Japan and are well accepted by patients.^30^, ^31^ Kampo medicines are probably chosen in Japan because OCPs and SSRIs are not easily accepted by the general public.

There have been some studies on the therapeutic efficacy of drospirenone‐containing OCPs for PMDD; however, based on the results of this study, some OB/GYNs opt for this drug, but most psychiatrists do not use it.^16^ SSRIs are considered the first choice for treatment, but OCPs are also effective, and prescribing OCPs would expand psychiatrists' treatment toolkit.^10^, ^32^ In Japan, there is a prevailing tendency for OB/GYNs to address PMS due to its predominantly physical nature, whereas patients with PMDD cases are usually considered to be managed by psychiatrists. However, as seen in Table 2, a considerable portion of psychiatrists (60%) advocated for the involvement of psychiatry in addressing PMS, while the majority of OB/GYNs (77.8%) believed PMDD management should fall within their domain. These findings underscore the necessity for both departments to encourage the involvement of a multidisciplinary team; this situation could be managed by both departments working closely together and leveraging their combined expertise with their familiarity of treatment procedures. Specifically, psychiatrists should be able to confidently prescribe OC/LEPs, while OB/GYNs should be equipped to administer SSRIs.

Many psychiatrists also selected anti‐anxiety agents (58.7%), and despite benzodiazepines having a history of being one among the clinically used agents for the treatment of PMS/PMDD, there are very few reliable studies evaluating their efficacy, and they should be considered only for adjunctive therapeutic option in intractable cases.^33^, ^34^

Regarding non‐pharmacological therapies, lifestyle guidance was the most common, followed by counseling, whereas CBT, said to be as effective as SSRIs, was less common in both departments.^35^ The effectiveness of CBT for PMS/PMDD is supported by existing evidence, and the CBT implementation rate for depression and anxiety in psychiatric clinics in Japan is relatively high at 37.9%.^36^ However, the adoption of a CBT approach for PMS/PMDD is not widespread in Japan; the establishment of a standardized protocol could likely promote its wider adoption as a treatment modality.

The survey revealed that many doctors use methods unique to Japan that are not included in the global guidelines for diagnosis and treatment. The existing evidence supports the use of psychological therapies and non‐pharmacological complementary and alternative approaches for patients with mild symptoms who refuse pharmacotherapy. When the symptoms are more severe and the patient consents, SSRIs and OCPs should be preferred, as they represent the most reliable evidence‐based treatments.

Differences were found in the proportion of male and female psychiatrists who responded to the survey. In our previous study, we showed that gender differences among Japanese OB/GYNs influence the choice of diagnosis and treatment of PMS/PMDD.^37^ Originally, there was a larger gender difference in the number of Japanese psychiatrists compared to OB/GYNs, which influenced the composition of the participants in this study, therefore even if the gender difference observed in psychiatrists affects diagnosis and treatment, it cannot be simply compared with that amongst the OB/GYNs. As the purpose of this study was to investigate the current state of diagnosis and treatment between psychiatrists and OB/GYNs, the gender difference amongst psychiatrists was not investigated.

This is the first study in Japan to present the current status of psychiatry in diagnosing and treating PMS/PMDD. However, it should be mentioned that this study has some limitations. First, the psychiatrists who responded to this survey were members of JAPC, an organization mainly composed of physicians working in clinics and excluding those working in hospitals, therefore these data might be biased, even after considering that treatment for PMS/PMDD is mainly provided in outpatient clinics. Additionally, even though 15.7% of JAPC members responded to the survey, it is deduced that the survey responses should be considered as data from those highly concerned about PMS/PMDD who are active in those treatments. Second, regarding the assessment methods, only self‐reported data were used. Moreover, it was not possible to confirm the frequency with which the departments in this study would be encountering patients with PMS/PMDD, and it is possible that doctors under‐ or over‐reported certain information. Because this study relied on retrospective reporting of typical practices, the accuracy of answers concerning diagnosing and treating PMS/PMDD could be improved if these practices were assessed prospectively. Finally, other types of health professionals who might be related to the assessment of PMS/PMDD, such as internists and family physicians, were excluded. It is also important to investigate the clinical practices of PMS/PMDD in other countries to assess the practices in other regions.

## CONCLUSIONS

Most psychiatrists as well as OB/GYNs in Japan based their diagnosis of PMS/PMDD on vague medical interviews rather than on a prospective monitoring method in accordance with the relevant diagnostic criteria. Moreover, our results also indicate that they do not treat patients based on appropriate evidence. With regard to treatment, there was a tendency for bias toward treatments in which their department specializes, which differs from treatments in accordance with global standard guidelines, and many treatments are unique to Japan, such as Kampo medicine. Collectively, our findings indicate that it is necessary to further educate psychiatrists and OB/GYNs about PMS/PMDD. Tailor‐made treatment regimens based on evidence‐based medicine need to be developed by assessing the needs of individual patients and appropriate use of a multidisciplinary approach that involves both psychiatrists and OB/GYNs as needed.

## AUTHOR CONTRIBUTION

All authors contributed to the study's conception, study design, and manuscript revision and approved the final manuscript. Kana Yoshimi analyzed the data and wrote the manuscript. Takashi Takeda was the main contributor to the study design and conception.

## CONFLICT OF INTEREST STATEMENT

Tamami Odai was supported by grants from Ibaraki Prefecture and Japan Agricultural Cooperatives of Ibaraki Prefecture. Tempei Otsubo has received personal fees from Viatris, Takeda Pharmaceutical, Otsuka Pharmaceutical, Sumitomo Pharma, Mochida, Meiji Seika Pharma, and Kyowa Pharmaceutical and grants from Eisai and Otsuka Pharmaceutical outside the submitted work. Masakazu Terauchi was supported by grants from Ibaraki Prefecture and Japan Agricultural Cooperatives of Ibaraki Prefecture, and lecture fees from Fuji Pharma Co., Ltd., Bayer Pharma Co., Ltd., Tsumura Co., Ltd., Otsuka Pharmaceutical Co., Ltd., and Astellas Pharma Inc. Takashi Takeda was supported by grants from Tsumura Co., Ltd. and Otsuka Pharmaceutical Co., Ltd. and received lecture fees from Tsumura Co., Ltd., Otsuka Pharmaceutical Co., and Bayer Pharma Co., Ltd. None of the funding agencies had any role in the study design or manuscript preparation. The authors report no other conflicts of interest regarding this work.

## ETHICS APPROVAL STATEMENT

This study was conducted in accordance with the principles outlined in the Declaration of Helsinki. The survey was anonymous and did not include any personal information.

## PATIENT CONSENT STATEMENT

Before completing the survey, all participants read the description of the study's purpose and agreed to participate in the study by providing online consent.

## CLINICAL TRIAL REGISTRATION

N/A.

## Data Availability

The data that support the findings of this study are available from the corresponding author upon reasonable request.
